# Psychodynamically Oriented Psychopharmacotherapy: Towards a Necessary Synthesis

**DOI:** 10.3389/fnhum.2019.00015

**Published:** 2019-01-31

**Authors:** Angela Iannitelli, Serena Parnanzone, Giulia Pizziconi, Giulia Riccobono, Francesca Pacitti

**Affiliations:** ^1^Department of Biotechnological and Applied Clinical Sciences, University of L’Aquila, L’Aquila, Italy; ^2^Psychoanalytical Centre of Rome (CPdR), Italian Psychoanalytical Society (SPI), Rome, Italy; ^3^International Psychoanalytical Association (IPA), London, United Kingdom; ^4^Psychiatry Unit San Salvatore Hospital, L’Aquila, Italy

**Keywords:** psychoanalysis, psychotropic drugs, integrated therapies, psychodynamic therapy, psychotic disorders

## Abstract

The discovery of psychoanalysis and of psychotropic medications represent two radical events in understanding and treatment of mental suffering. The growth of both disciplines together with the awareness of the impracticality of curing mental suffering only through pharmacological molecules—the collapse of the “Great Illusion”—and the experience of psychoanalysts using psychotropic medications along with depth psychotherapeutic treatment, have led to integrated therapies which are arguably more effective than either modality alone. The authors review studies on the role of pharmacotherapy with psychoanalysis, and the role of the analyst as the prescriber. The psychotic disorders have specifically been considered from this perspective.

## Introduction

“[‥.] They don’t realize we’re bringing them the plague” whispered Freud to Jung sailing into New York Harbor, on August 21, 1909 (Lacan, 1996). It is a fact that psychoanalysis, as formulated by Sigmund Freud halfway between two centuries, brought the “plague” not only to the United States, but the entire world, representing one the most influential intellectual medical movements in the whole of human history. Expanding its observations from human suffering to societal interaction, psychoanalysis would have a profound impact on innumerable cultural products of human creativity such as literature, art, and cinema, and even other epistemological sciences (Tobin, 2011; Buckley, 2012; Scull, 2014; De Fiore, 2017). Despite this, psychoanalysis has undergone a storm of criticism over the last century. Detractors have pointed out that it lacks scientific credibility and that the extent to which it is evidence-based is weak and patchy. However, more recently, there have been a rise in the number of high-quality randomized controlled trials and neuroscientific studies that have provided support for many of Freud’s clinical intuitions, leading to the rebuttal of the initial accusations of psychoanalysis as not being scientific (Solm, 2008; Brockman, 2011; Panksepp and Solms, 2012; Salone et al., 2016). Indeed, nowadays, psychoanalysis represents a “living” reality that cannot be ignored, with millions of patients treated worldwide from many “societies” scattered across many countries, including Russia, Iran, and China.

On the other hand, at the beginning of the 1950s, the fortuitous discovery of what would become the first psychotropic drugs—chlorpromazine, iproniazid, imipramine, and chlordiazepoxide—has led to the development of new pharmacological classes, such as antidepressants, antipsychotics, mood stabilizers, and anxiolytics (Ban, 2001). This has allowed the curing of millions of people worldwide. Furthermore, the broadening of knowledge regarding the pathogenesis of various mental illnesses has contributed to the closure of mental asylums in many countries, including Italy (Scull, 2010).

For many years, psychoanalysis and psychopharmacology have followed parallel paths. The former, aimed at defending its own epistemology and its own growth—generally by its power apparatus—with a particular propensity for theorization; the latter, aimed at exploring the full clinical potential of psychotropic drugs—with its undoubted economic interests—but also genuinely aimed at understanding their specific mechanisms of action and thus the alterations underlying mental disorders (Scull, 2010). The growth of both disciplines together with the awareness of the impracticality of curing mental suffering only through pharmacological molecules, emergence of new forms of psychic suffering, and mutually empowering dialog between neuroscience and psychoanalysis have finally led to the development of so-called integrated therapies pointing to greater effectiveness than the use of psychoanalysis or pharmacotherapy alone. Thus far, there have been only a few studies on the results of combination/comparison and the interaction of proper long-term psychoanalysis with drug therapy. However, in the past two decades, there have been a significant number of studies that have investigated this question for other forms of psychotherapy (e.g., cognitive-behavioral therapy, interpersonal therapy, family therapy) and that have demonstrated, especially for depression and anxiety disorders, a pronounced effectiveness of integrated treatment over psychotherapy or drug therapy alone (Cuijpers et al., 2014; Aaronson et al., 2015).

Given that psychoanalysts today, partially due to the economic crisis, are likely to treat patients who are on drug therapy at the beginning of their analytic process or may need the use of drugs at some point during their analysis, the purpose of our article will be to review existing research concerning the combination of psychoanalysis and pharmacotherapy. In this context, a specific focus will be given to the psychotic dimension, as stated by Holmes (2012), fully exemplifying in miniature, the rise, fall, and tentative rebirth of a modern psychoanalysis. Our hope is that a better understanding of this alternative/integrated approach will benefit the patient and will finally enhance communication between the frequently competing worlds of psychoanalysis and psychiatry.

## Pharmacotherapy in Psychoanalysis

Reviewing the psychoanalytic literature on the use and efficacy of combined psychoanalysis and drug therapy reveals considerable controversy that is often not openly addressed (Cabaniss and Roose, 2005; Gorman, 2016).

Systematic studies have highlighted how psychoanalysts may present difficulties in shifting from a dynamic model of mind to a phenomenologically based diagnostic system, which is more necessary to provide proper pharmacological treatment (Baumeister and Hawkins, 2005; Cabaniss and Roose, 2005; Purcell, 2008). In addition, regardless of the diagnosis, psychoanalysts may feel that they have failed if they need to “resort” to medication, as psychoanalysis alone is not sufficient in treating a patient (Cabaniss and Roose, 2005). In traditional psychoanalytic practice, the use of medication has always been considered a deviation from the standard analytic process, to be prescribed only if necessary, and to be stopped as soon as possible to return to the analysis of transference. After all, medication reduces the current turmoil, but does not address the underlying psychological factors of that turmoil or maladaptive defenses that predispose patients to symptoms. On the other hand, psychoanalysis can help individuals identify sources of psychic pain, with the patient eventually gaining greater emotional and behavioral adaptability (Normand and Bluestone, 1986; Loeb and Loeb, 1987; Mintz, 2006; Press, 2008; Kaplan, 2014).

Some authors have argued that reducing emotional pain with medication might decrease motivation for the analytic process leading to premature termination or pseudo-successful outcome, thus making psychopathology more likely to recur. Another concern has been that the prescription of psychotropic medication by the analyst would interfere with the phenomenon of transference/countertransference (Kubie, 1958; Normand and Bluestone, 1986; Gibbons et al., 2008; Kaplan, 2014). In fact, patients who are interested in self-exploration tend to continue with psychoanalysis regardless of whether they have been prescribed medication. In contrast, many patients receiving psychopharmacological treatment express the wish to learn about and master the conflicts that cause the symptoms with the hope of making medication unnecessary in the future (Kaplan, 2014). Regarding the possible interaction between medication and transference/countertransference, as stated by some authors, whether it occurs mostly depends on how well the analyst is able to recognize and manage it (Normand and Bluestone, 1986).

These concerns aside, an important consideration that should be addressed involves the concept of the “psychosomatic,” the interaction of mind and body, and its role in the causation and curing of diseases. The medical literature has increasingly accepted the importance of psychological factors in the onset and progress of all illnesses. Conversely, many cases of diseases considered purely “mental” have been shown to have physical, possibly remediable, causes (e.g., epilepsy). Almost 50% of psychiatric patients report that fatigue, muscle pain, headache, abdominal pain, and backache are associated with sadness or panic as primary manifestations of anxiety and depression. Depression is indeed increasingly being recognized as a comorbid disorder in patients with severe and chronic medical conditions and pain syndromes. Following this line of thought, emotional disorders should involve a combined treatment approach. Thus, the use of medication should be maintained as long as necessary to control the symptoms, in the case of severe mental illnesses, possibly endangering the patient’s life or continuation of the therapy (Gochfeld, 1978; Biondi, 1995; Iannitelli and Tirassa, 2015).

At present, although still considered almost a “nonsanctioned maneuver” that is rarely debated in most psychoanalytic circles, even in the presentations of cases where drugs have effectively been used, there is a widespread acceptance in clinical practice of a combined use of psychoanalysis and drug therapy, mainly antidepressants (Mandell, 1968). This is in line with studies showing that between 30 and 50% of patients currently entering psychoanalysis present with an Axis I mood and/or anxiety disorder (Vaughan et al., 1997).

As early as 1962, Mortimer Ostow, a psychoanalyst trained in neurology, pointed to the utility of using psychotropic drugs during the course of psychoanalysis (Kandel, 1999). In 1977, a panel discussion at the meeting of the American Psychiatric Association, which included several analysts, agreed that psychotropic drugs “must be used” in certain circumstances. Indeed, the combination of psychoanalysis with drug therapy offers new possibilities for the treatment of seriously ill patients. This applies in particular to patients more vulnerable to stress that could be treated analytically by using medication to prevent serious depression, psychotic decompensation, or destructive behavior (Normand and Bluestone, 1986; Ramos, 2013). In a survey of members of the American Academy of Psychoanalysis, 90% of respondents revealed they were prescribing medications. Similarly, in a study of psychoanalytic candidates’ training cases at the Columbia University Centre, psychoanalysis had been combined with drug therapy in 29% of cases (Roose and Stern, 1995).

Regarding outcome studies, the few that have been performed thus far (mostly case studies, but also clinical and randomized controlled studies) suggest that the combination of psychoanalysis and medication may be superior for the treatment of mood and anxiety disorders. However, most of these studies have small sample sizes and involve only short-term psychotherapy (Gorman, 2016). There is no question that for this idea to be taken seriously, larger-scale studies, rigorous controls, and the cooperation of all therapists and patients are required. The impetus to perform such work certainly comes from significant neuroscience data suggesting several routes by which the two treatments could be synergistic including the stimulation of hippocampal neurogenesis, epigenetic regulation, dendritic remodeling, enhanced prefrontal cortical control of limbic activity, and activity at specific neurohormonal and/or neurotransmitter targets (Solm, 2008; Gorman, 2016; Salone et al., 2016).

## The Role of the Analyst as Prescriber: Combined or Split Treatments?

As summarized by Awad (2001), in psychoanalysis there are usually three ways of prescribing medication. The first method is for a medical analyst to diagnose a condition that requires medication, to prescribe it, and be responsible for monitoring dosage and side effects. The second option is to refer the patient to a psychiatrist, who prescribes and monitors the medication. The third is to refer the patient for a consultation to eventually introduce medication and to take responsibility for prescribing and monitoring it.

A number of factors, such as the analytic technique chosen, the patients’ diagnosis and personality, and/or the lack of a sufficient medication expertise may contribute to the analyst’s decision to split treatment rather than to prescribe medication (Lebovitz, 2004; Olesker, 2006; Kaplan, 2014). Some analysts argue in favor of sending their patients to a psychopharmacologist for consultation in order to preserve an analytic stance and avoid contamination of the transference (Wylie and Wylie, 1995). Others opt for referring their patients to a psychopharmacologist in order to avoid inadequate pharmacological treatment since answering patients’ questions about the effects/side effects of their medication may lead to a disruption of the analytic process and a forsaking of technical neutrality (Adelman, 1985; Yudofsky, 1991; Vlastelica, 2013; Sandberg, 2014; Salone et al., 2016). Still others prefer to administer the pharmacological treatment and claim that the discussion regarding medications and symptomatic changes may become a positive part of the complex fabric of the analytic relationship (Kandel, 1999; Greene, 2001; Glucksman, 2006; Scull, 2010). Furthermore, referring patients to others may be viewed negatively by the analysands, not to mention the fact that the shared responsibility may become an arena of conflict and struggle for control (Awad, 2001).

The fact that patients often fail to respond to medication for several reasons including ongoing use of problematic defense mechanisms, hidden use of alcohol and/or other drugs, and nonadherence, is a further claim in favor of a supportive therapeutic alliance with the prescribing doctor (Kaplan, 2014). In line with this statement, an in-depth knowledge of the patient’s unique biopsychosocial aspects and psychodynamics, integrated with a variety of attachment theoretical concepts, has been indicated as leading to a more efficient pharmacological prescription, and in the case of non-adherent patients with dismissing attachment behaviors, helping them to receive the recommended pharmacological treatment. It should be noted that non-specific factors, such as adherence to treatment protocols together with a therapeutic alliance and the therapist’s competence significantly contribute to treatment outcomes and may account for more of the variance than the specific treatment approach (Chatoor and Krupnick, 2001). In a more holistic perspective, this further confirms the importance of a strong analytic/medical-patient relationship (Alfonso, 2009; Ramos, 2013).

## Psychodynamic Psychotherapy and Psychopharmacology of Mental Disorders—A Specific Focus on Psychosis

In the 1950s, mental health professionals shared a short-lived hope that drugs could definitively cure mental suffering. However, this did not occur and despite the positive effects of medication on a lot of patients, many symptoms remained untreated. In some cases patients experienced serious side effects. Against this backdrop, the mental health community started to turn its attention to social interventions and psychotherapy (Tai and Turkington, 2009; Brus et al., 2012; Iannitelli, 2014). Following this line of thought, research outcomes and clinical practice have encouraged psychodynamic psychotherapy, positioning such treatment among recommendations for treating various mental disorders, including patients with psychotic disorders (Ivezić et al., 2017).

Regarding schizophrenia, Freud and his more orthodox followers felt that patients with schizophrenia were not suitable for psychoanalysis. However, numerous dissenters have enthusiastically advocated for their treatment (Brus et al., 2012). For decades, the efficacy in schizophrenia of combined treatment with psychodynamic psychotherapy and drug therapy has been questioned.

In their study with 228 state hospital patients with schizophrenia, May et al. (1981) reported that both patients treated only with medication and those treated with individual supportive psychodynamic psychotherapy in conjunction with antipsychotics had a reduced length of hospitalization and tended to be readmitted less and for shorter period than patients who received only supportive psychodynamic psychotherapy.

After a careful analysis of all randomized trials of individual psychodynamic psychotherapy for people with schizophrenia, Malmberg and Fenton (2001) highlighted the superior effectiveness of medication compared to psychodynamic psychotherapy in achieving a hospital discharge, assuming that psychotherapy had a beneficial effect, even if the data were sparse, only for patients given additional medication in the 12 months to 3 years after discharge. In support of these findings, Michels (2003) stated that even if the psychoanalytic approach was able to offer a better understanding about how patients cope with schizophrenia, it does not tell us much about the disorder itself, and in general has little special relevance to the disorder. He finally defined schizophrenia as a relative contraindication to psychoanalytic treatment and underlined the importance of antipsychotic medication not so much for its sedative effects but for its neuropsychological benefits.

However, other authors have expressed different positions, and some have claimed that psychoses and schizophrenia can no longer be seen as chronic deteriorating conditions, since recovery is possible in many patients with psychosis or schizophrenia treated with approaches that focus on the primacy of psychoanalytically oriented psychotherapeutic intervention (Gibbs, 2007). One of the central tenets of psychotherapy is the therapist and their experience, which plays a significant role in the treatment outcome. Specific therapists’ characteristics, such as their attitudes, intellectual and therapeutic skills, and ability to deal with stress or convey acceptance and compassion, have been reported to indirectly influence the treatment outcome (Karon, 1981). Frank and Gunderson’s (1990) investigation of the role of the therapeutic alliance in the treatment of patients with schizophrenia also revealed that a good alliance with therapists within the first months was strongly correlated to the treatment course and outcomes, specifically with respect to patients’ greater acceptance of both psychotherapy and pharmacological treatments and reduced medication use. Based on the Danish National Schizophrenia Project, some studies (prospective, comparative, longitudinal multi-site investigations) suggest the greater efficacy in the treatment of patients with schizophrenia with individual supportive psychodynamic psychotherapy in addition to treatment as usual compared to treatment as usual alone (Rosenbaum, 2009; Rosenbaum et al., 2012). Patients improved significantly during the 2 years of treatment with moderate to strong effect sizes on positive and negative symptoms, general symptom level, and social function. In line with these findings, other studies found support for improved symptomatology along with changes in some cognitive/social functioning, and quality of life of patients with schizophrenia receiving long-term (up to 3 years) psychodynamic group therapy in addition to regular antipsychotic treatment (Restek-Petrović et al., 2014; Pec et al., 2018).

Taken together, all these studies suggest the importance of patients receiving integrated psychoanalysis and pharmacological treatment, specifically suggesting how psychiatric and psychoanalytic principles are intimately linked together and useful in understanding patients’ illness and treating them successfully. In line with these findings, it is our hope that the clinical potential of the theoretical and practical alliance between psychoanalysis and neuroscience will no longer be underestimated and that it instead will be further investigated.

## Author Contributions

All authors have contributed significantly and are in agreement with the content of the manuscript.

## Conflict of Interest Statement

The authors declare that the research was conducted in the absence of any commercial or financial relationships that could be construed as a potential conflict of interest.
